# A systematic analysis of *C5ORF46* in gastrointestinal tumors as a potential prognostic and immunological biomarker

**DOI:** 10.3389/fgene.2022.926943

**Published:** 2022-08-05

**Authors:** Yuhong Jiang, Xiaobo Wang, Lun Li, Jun He, Qianqian Jin, Dongju Long, Chao Liu, Weihan Zhou, Kuijie Liu

**Affiliations:** ^1^ Department of Gastroenterology, The Second Xiangya Hospital of Central South University, Changsha, Hunan, China; ^2^ Department of Breast-Thyroid Surgery, The Second Xiangya Hospital of Central South University, Changsha, Hunan, China

**Keywords:** C5ORF46, gastrointestinal tumors, prognosis, tumor microenvironment, immune infiltration, immunotherapy

## Abstract

**Background:** Chromosome 5 open reading frame 46 (*C5ORF46*), also known as antimicrobial peptide with 64 amino acid residues (*AP-64*) and skin and saliva-secreted protein 1 (*SSSP1*), belongs to the family of open reading frame genes and encodes a small exosomal protein. *C5ORF46* has been implicated in antibacterial activity and associated with patient prognosis in pancreatic cancer, colorectal cancer, and stomach cancer. These findings highlight the importance of *C5ORF46* in gastrointestinal (GI) tumor inception and development. However, the prognostic and immunological value of *C5ORF46* in human GI tumors remains largely unknown. In this study, we sought to explore the potential value of *C5ORF46* in GI tumor prognosis and immunology.

**Method:** RNA sequencing (RNA-seq) was performed on the tumor and tumor-adjacent normal samples we collected to identify potential target genes for GI tumors. Apart from our RNA-seq data, all original data were downloaded from The Cancer Genome Atlas (TCGA) database and integrated *via* Strawberry Perl (v 5.32.0) and R (v 4.1.1). The differential expression of *C5ORF46* was examined with Oncomine, Tumor Immune Estimation Resource (TIMER), Gene Expression Profiling Interactive Analysis (GEPIA), Cancer Cell Line Encyclopedia (CCLE), the Human Protein Atlas (HPA) and TCGA databases. The c-BioPortal database was used to investigate the genomic alterations of *C5ORF46*. The effect of *C5ORF46* on prognosis and clinical phenotypes was explored *via* bioinformatics analyses on the TCGA and GEPIA databases. We used the bioinformatics analyses based on the TCGA database to analyze tumor mutational burden (TMB), microsatellite instability (MSI), tumor immune cell infiltration, and the correlations between *C5ORF46* expression and several immune-related genes. Kyoto Encyclopedia of Genes and Genomes (KEGG) pathway enrichment analysis was carried out *via* the DAVID website and presented as bubble charts using ShengXinRen online tools. Gene set enrichment analysis (GSEA) was performed using R scripts based on data downloaded from the GSEA website. Immunohistochemistry (IHC) was used to validate the expression of *C5ORF46* in GI tumors.

**Results:** The results of our RNA-seq data indicated a critical role for *C5ORF46* in colon carcinogenesis. Consistently, we demonstrated that *C5ORF46* was highly expressed in tumor tissues compared to normal tissues in human GI tumors. Moreover, a strong correlation was observed between *C5ORF46* expression levels and patient prognosis, staging, TMB, MSI, and immune cell infiltration. Further, *C5ORF46* presented as an important regulator in the tumor microenvironment (TME) and was active in the regulation of cancer immune functions. *C5ORF46* is significantly correlated with genes regulating inflammation and immune responses.

**Conclusion:** *C5ORF46* may serve as a biomarker for GI tumor prognosis and immunology. *C5ORF46* could be a novel target for GI tumor immunotherapy.

## Introduction

According to the International Agency for Research on Cancer and the World Health Organization data published in 2020, an estimated 19.3 million new cases of cancer were diagnosed and almost 10 million cancer-related deaths were reported worldwide cross 36 types of cancers. Of these, GI tumors account for 26.4% of cancer incidence and 36.3% of cancer-related mortality (Sung et al., 2021). Although GI tumors account for a large proportion of cancer-related deaths, they are hard to treat. This is largely because of late diagnosis at an advanced stage of the condition when curative resections are not suitable (Valladares-Ayerbes et al., 2008). For GI tumor patients, options for radical surgery are limited and chances of survival are poor. Further, recurrence and metastases frequently occur (Valladares-Ayerbes et al., 2008), and standard therapies are limited in treating refractory GI tumors (Kirk, 2012). In recent years, advances in genomic, transcriptomic and epigenomic assays have enabled researchers to use new technologies, such as next-generation sequencing, to study the genetic characteristics of GI tumors, which significantly contribute to cancer diagnosis and prognosis (Li et al., 2021). There remains an urgent need to identify effective prognostic biomarkers to better diagnose and treat GI tumors.

*C5ORF46* is a 7.2 kDa mammal-specific exosomal protein belonging to the family of open reading frame genes (Harney et al., 2019). Although not every open reading frame has a corresponding functional protein product, *C5ORF46* is a protein encoding gene which has been shown to encode an antimicrobial peptide (AMP) and to exhibit antibacterial activity against Gram-negative bacteria. *C5ORF46* is also termed *AP-64* (Zhong et al., 2021, 46) and *SSSP1* for its distribution across the body. *C5ORF46* is predicted to be located in extracellular exosomes based on Gene Ontology (GO) annotation (Alliance of Genome Resources, 2022). A recent study detected *C5ORF46* in human plasma and revealed *C5ORF46* to be a previously-uncharacterized small human plasma protein that may be associated with lipid homeostasis (Harney et al., 2019). Several reports implicate *C5ORF46* as having a pro-tumoral role with possible prognostic value in the development of pancreatic adenocarcinoma (PAAD) (Makler and Narayanan, 2017), colon adenocarcinoma (COAD) (Chen et al., 2021) and stomach adenocarcinoma (STAD) (Cheng et al., 2020), which underline the value of studying *C5ORF46* in GI tumors (Makler and Narayanan, 2017; Cheng et al., 2020). However, with limited evidence, the role of *C5ORF46* in cancer development remains unexplained.

Researchers have recently made substantial efforts to build immunotherapeutic weapons to fight tumor development, for example, immune checkpoint inhibitors (ICIs). These ICIs are a new family of immunotherapeutic medicines with the potential to activate the immune system. They have emerged as substantially transformative in the treatment of malignancies, including those in the gastrointestinal tract (Ruiz-Bañobre and Goel, 2019). The emergence of these new drugs unveiled the substantial efforts in recent years that have been put into the research of TME, an immunosuppressive compartment contributing to tumor development. (Pitt et al., 2016; da Silva et al., 2019). The ICIs, for example, ipilimumab (anti-CTLA-4), nivolumab and pembrolizumab (anti-PD-1), can target the CTLA-4 and PD-1 receptors to relieve their inhibition of T cell infiltration in the TME (Ruiz-Bañobre and Goel, 2019). However, the clinical responses of cancer patients to immunotherapy were not optimal. Evidence indicates that certain genetic or genomic markers, such as PD-L1 expression (Taube et al., 2014), mismatch-repair status (Le et al., 2015), tumor mutational burden (Goodman et al., 2017), and tumor aneuploidy (Davoli et al., 2017; Li et al., 2019), are linked to cancer immunotherapy responses. Thus, new targets are required to increase the efficacy of cancer immunotherapy.

In this study, we used multiple databases to analyze differences in *C5ORF46* expression levels between GI tumor tissues and tumor-adjacent normal tissues, and in various cell lines. Using TCGA datasets, we assessed the prognostic validity of *C5ORF46* in seven GI tumors. Across these seven tumors, we considered the relationship between *C5ORF46* expression levels and TMB, MSI, TME, and immune cell infiltration. Further, we used correlation and enrichment analysis of *C5ORF46* and immune-related genes to study the biological functions of *C5ORF46* in GI tumors. To further validate our results, we conducted IHC staining to examine the expression of *C5ORF46* in GI tumor samples. Our results reveal that *C5ORF46* is a potential prognostic and immunological marker for GI tumors.

## Materials and methods

### Data and software availability

Apart from our RNA-seq data, all source data were downloaded from TCGA, which contains 11,069 samples from 33 different types of cancer, *via* the University of California Santa Cruz (UCSC) Xena browser (https://xena.ucsc.edu/). The gene expression data of normal tissues were extracted from the Genotype-Tissue Expression (GTEx) (https://gtexportal.org/home/datasets). *C5ORF46* gene expression data were extracted from these downloaded datasets and plotted into a data matrix for further analysis using Strawberry Perl (version 5.32.1.1, http://strawberryperl.com/). The R 4.1.1 program was used to combine the original data and to confirm the results of the website database analysis. The online website tools used in this analysis are outlined below.

### Ethics statement

This study was approved by the Ethics Committee of the Second Xiangya Hospital [2018(Yan149)].

### RNA sequencing analysis and identification of *C5ORF46*

Two colorectal cancer patients who had undergone tumor resection surgery in the Second Xiangya Hospital, Hunan, China, were randomly selected for the study. Samples from their colorectal cancer tissue and tumor-adjacent normal tissue were collected and preserved in liquid nitrogen for RNA extraction. Total RNA for each sample was extracted using TRIzol Reagent (Invitrogen, Carlsbad, CA, United States).

The total amount and integrity of RNA was detected using Agilent 2100 bioanalyzer. The cDNA library was constructed by enriching mRNA with the polyA tail from total RNA by Oligo (dT) magnetic beads, and randomly fragmenting the mRNA obtained with divalent cations in a Fragmentation Buffer. The first strand of cDNA was synthesized in the M-MuL V reverse transcriptase system using random oligonucleotides as primers and fragmented mRNA as templates. The second strand of cDNA was synthesized under the DNA polymerase I system using dNTPs from the RNaseH-degraded RNA strand. The purified double-stranded cDNA was end-repaired, A-tailed, and connected to a sequencing adapter. AMPure XP beads were used to screen cDNAs of approximately 370–420 bp followed by PCR amplification. AMPure XP beads were again used to purify the PCR products. Finally, a library was obtained, after which a Qubit 2.0 Fluorometer was used for preliminary quantification. The library was diluted to 1.5 ng/ul, and an Agilent 2100 bioanalyzer was used to detect the insert in the library. After confirming that the insert size was as expected, qRT-PCR was performed to accurately estimate the effective concentration of the library (ideally greater than 2 nM) to guarantee the library’s quality. Following the qualification of the library check, the various libraries were pooled according to the requirements of effective concentration and target data volume, and Illumina sequencing was carried out. Clean reads were generated by removing raw reads with an adapter or any N (i.e., base information cannot be determined), and low-quality bases (i.e., reads with bases that have Qphred ≤ 20 that account for more than 50% of the entire read length). Q20, Q30, and GC ratios were calculated as the indicator to evaluate clean reads. Reads were aligned against the reference genome using HISAT2 v2.0.5. The raw data in this article have been deposited in Gene Expression Omnibus (GEO) and are accessible through GEO accession number GSE200427. Differential expression analysis was performed using the ‘limma’ package in R software. Our criterion was set as log2FC > 1 and *p*-value < 0.05 for identifying significantly upregulated genes in GI tumors.

Five hundred differentially-expressed genes (DEGs) were downloaded from the public database GEPIA (http://gepia2.cancer-pku.cn/#survival) with a selection of the “COAD” dataset, setting “OS” as methods, and “quartile” as the group cutoff value. Subsequently, we visualized the intersected DEGs of the two datasets using the “VennDiagram” package in R software.

### Expression landscape analysis

The levels of *C5ORF46* gene expression in seven GI tumors were examined in the Oncomine database (https://www.oncomine.org/resource/login.html) (Rhodes et al., 2007), the TIMER database (https://cistrome.shinyapps.io/timer/) (Li et al., 2017), and the GEPIA database (http://gepia2.cancer-pku.cn/#analysis) (Tang et al., 2017). In the Oncomine database, the *p*-value was set at 1e^−4^, the fold change was set at 2, and the gene ranking was set at 10%. The CCLE database was used to download data from each tumor cell line (https://portals.broadinstitute.org/ccle/). Apart from the online databases, expression of *C5ORF46* was evaluated in our downloaded TCGA and GETx datasets, where *C5ORF46* expression levels were compared between tumor and tumor-adjacent normal tissues in GI tumors. Expression data were Log2 transformed, and two sets of t-tests were run. *p*-value < 0.05 was regarded as sufficient to indicate a differential expression between tumor and tumor-adjacent normal tissues. The R programming language was used to analyze the data and R package “ggpub” was utilized to construct box plots.

### Immunohistochemistry and evaluation of staining intensity for *C5ORF46*

IHC pictures of *C5ORF46* protein expression in tumor and normal liver tissues were downloaded from the Human Protein Atlas (HPA) database (http://www.proteinatlas.org/) and investigated to assess differences in *C5ORF46* expression at the protein level (Antibody: HPA079692). To further provide clinical validity of *C5ORF46* expression differences in GI tumors, we performed IHC using clinical samples of seven GI tumors. Forty-two tissue sections (three samples for each GI tumor and tumor-adjacent normal tissue) were collected to conduct IHC experiments. Paraffin-embedded sections were dewaxed with xylene and rehydrated through ethanol. Then the sections were incubated with 3% H2O2 for 20 min to block endogenous peroxidase and then treated with 1 mM EDTA to retrieve the antigen. Sections were incubated with 1:50 diluted anti-*C5ORF46* antibody (HPA079692, Atlas Antibodies, Sweden) at 4°C overnight. After incubation with a 2-step plus Poly-HRP Anti-Mouse/Rabbit IgG Detection System (PV-9000, Zhongshan Jinqiao Biotechnology Company, Beijing, China), the sections were visualized with the diaminobenzidine (DAB; Zhongshan Jinqiao Biotechnology Company, Beijing, China), counterstained with hematoxylin, and dehydrated. Sections were scanned using a Zeiss microscope. Staining intensity was measured and quantified using the ImageJ plug-in “IHC Toolbox” and GraphPad Prism version 7 software, respectively. *p*-value < 0.05 was considered as significant. (*p < 0.05, **p < 0.01, ***p < 0.001).

### Genomic alterations

The cBio Cancer Genomics Portal (c-BioPortal) (http://cbioportal.org) database was used to explore *C5ORF46* genomic alterations in the seven GI tumors (Gao et al., 2013).

### Prognostic and clinical phenotypic analysis

For each sample obtained from the TCGA, survival and clinical phenotypic data were extracted. To investigate the link between *C5ORF46* expression and patient prognosis, three markers were chosen: overall survival (OS), disease-free interval (DFI)/disease-free survival (DFS), and progression-free interval (PFI). A survival analysis (*p*-value < 0.05) was performed for each cancer type using the Kaplan-Meier method and the log-rank test. The R packages “survival” and “survminer” were used to create survival curves. Cox analysis was used to assess the connection between *C5ORF46* expression and survival in GI tumors. Forest plots were created using Sangerbox online tools (http://sangerbox.com/AllTools?tool_id=9730908). We used the GEPIA database to ascertain if there was a link between *C5ORF46* expression and OS or DFS in seven GI tumors, including cholangiocarcinoma (CHOL), colon adenocarcinoma (COAD), esophageal carcinoma (ESCA), liver hepatocellular carcinoma (LIHC), pancreatic adenocarcinoma (PAAD), rectum adenocarcinoma (READ) and stomach adenocarcinoma (STAD).

The relationship between *C5ORF46* expression and two clinical characteristics, tumor stage and patient age, was investigated. The patients were split into two groups, with a cutoff age of 65 years. The R packages “limma” and “ggpubr” were used to conduct clinical phenotypic correlation analysis with *p*-value < 0.05 considered significant.

### Association analysis of *C5ORF46* with tumor mutational burden and microsatellite instability

Tumor mutational burden (TMB) quantifies the total number of mutations present in a tumor specimen. Microsatellites are short and repetitive DNA sequences randomly spread throughout the genome, and microsatellite instability (MSI) is formed by genetic alteration or sporadic epigenetic silencing, resulting in nucleotide insertions or deletions in microsatellite regions in the process of DNA replication. TMB and MSI both emerged as indicators of immune responses to predict cancer prognosis (Ratti et al., 2018; Choucair et al., 2020). TMB scores were calculated using a Perl script and rectified by dividing by the total length of exons. MSI scores were calculated for all samples using somatic mutation data from TCGA (https://tcga.xenahubs.net). Spearman’s rank correlation coefficient was used to examine the association between *C5ORF46* expression and TMB or MSI with *p*-value < 0.05 considered significant.

### Relationship between *C5ORF46* expression and immunity

Immune scores and stromal scores were calculated for each GI tumor by applying the Estimation of Stromal and Immune Cells in Malignant Tumor Tissues Using Expression Data (ESTIMATE) algorithm. The relationship between *C5ORF46* expression and these two scores was evaluated using the R software packages ‘estimate’ and ‘limma’. |R|≥0.3 and *p*-value <0.05 were considered significant.

We calculated the relative scores for 22 immune cells using CIBERSORT in the seven GI tumors. The R packages “ggplot2,” “ggpubr,” and “ggExtra” were used to examine the correlations between *C5ORF46* levels and each immune cell infiltration level in the GI tumors. |R|≥0.3 and *p*-value <0.05 were considered significant.

We used the R package “limma” to perform a correlation study of *C5ORF46* and immune-related genes, which included immune checkpoint genes, chemokine and chemokine receptor genes, antigen processing and presentation genes, interferon genes, and interleukin genes, downloaded from The Immunology Database and Analysis Portal (ImmPort) database (https://www.immport.org/shared/genelists). The R packages “reshape2” and “RColorBreyer” were used to visualize the results. |R|≥0.3 and *p*-value <0.05 were considered significant.

### Kyoto encyclopedia of genes and genomes pathway enrichment analysis

We used the GEPIA website (http://gepia2.cancer-pku.cn/#similar) to search for the top 300 genes that display similar expression patterns to *C5ORF46* in each GI tumor type, and then performed a KEGG pathway enrichment analysis on these genes using the DAVID website. We created bubble charts using ShengXinRen online tools to separately examine the pathway distribution. *p*-value < 0.05 was considered significant.

### Functional enrichment analysis

A GSEA was performed to investigate the biological activities of *C5ORF46* in GI tumors. We downloaded gene sets of GO biological processes (BP) and KEGG from the official GSEA website (http://www.gsea-msigPdb.org/gsea/downloads.jsp). The R packages “limma,” “org.Hs.eg.db,” “clusterProfiler,” and “enrichplot” were used to perform functional analyses. *p*-value < 0.05 was considered significantly enriched in the context of GSEA.

## Results

### Identification of *C5ORF46*

To better understand the molecular mechanisms of GI tumors, RNA-seq was performed using tumor and tumor-adjacent normal tissues from colorectal cancer patients. Processing of the raw RNA-seq data was described in the Method section above, and the following analysis was carried out based on the high-quality clean reads obtained. We compared the gene expression profile of tumor tissues with that of tumor-adjacent normal tissues. A total of 2,438 DEGs were sorted after limma analysis (*p* < 0.05). We set the threshold of log2FC > 1 to screen for genes highly expressed in tumor tissues compared to tumor-adjacent normal tissues, from which we obtained a total of 1,354 genes. To further verify our own RNA-seq results in public databases, we intersected the 1,354 genes with the 500 most differential survival genes in COAD downloaded from GEPIA. A total of 31 DEGs were finally obtained (Table 1). A Venn diagram was generated to show the intersecting genes (Supplementary Figure S1A). Among these genes, we noticed that *C5ORF46* was approximately 64-fold differentially expressed (log2FC = 6.18, *p* = 0.00071), which presented as the most differentially expressed one. Similarly, previous studies suggested a pro-tumoral and prognostic role of *C5ORF46* in several GI tumors, including PAAD (Makler and Narayanan, 2017), COAD (Chen et al., 2021), and STAD (Cheng et al., 2020). We postulated that *C5ORF46* may be a potential prognostic marker for human GI tumors.

**TABLE 1:** The intersected 31 differentially-expressed genes (DEGs) arranged by the value of log2FC.

Gene name	log2FC	*p*.Value
*C5ORF46*	6.18114	0.000713
*ELFN1-AS1*	5.39152	0.000549
*ITGBL1*	5.268365	0.002379
*AMH*	4.489819	0.016339
*TNNT2*	3.810652	0.01554
*NPM1P26*	3.525849	0.004526
*ANKRD18A*	3.319547	0.011707
*CCDC144NL-AS1*	3.123276	0.00393
*GDF15*	2.948614	0.013239
*CENPE*	2.89916	0.024246
*KCNQ1OT1*	2.893066	0.006425
*HMMR*	2.878511	0.003772
*SPHK1*	2.855175	0.040644
*TAS2R20*	2.523163	0.024483
*CDC25C*	2.45107	0.01754
*CDCA2*	2.3045	0.010448
*BMS1P1*	2.178506	0.049877
*GABRE*	1.955283	0.030682
*TIGD1*	1.810477	0.029758
*PCSK4*	1.698623	0.02652
*LENG8-AS1*	1.654147	0.030829
*KPNA2*	1.6344	0.00983
*ZNF37BP*	1.617392	0.044533
*MYO6*	1.565395	0.031324
*RRP12*	1.498251	0.044351
*RPL32P3*	1.496111	0.039087
*UACA*	1.355406	0.019206
*TIMP1*	1.344664	0.018463
*PHF14*	1.324854	0.011178
*ARHGAP4*	1.28956	0.032175
*DLD*	1.025713	0.042458

### Expression patterns of *C5ORF46*

**FIGURE 1:**
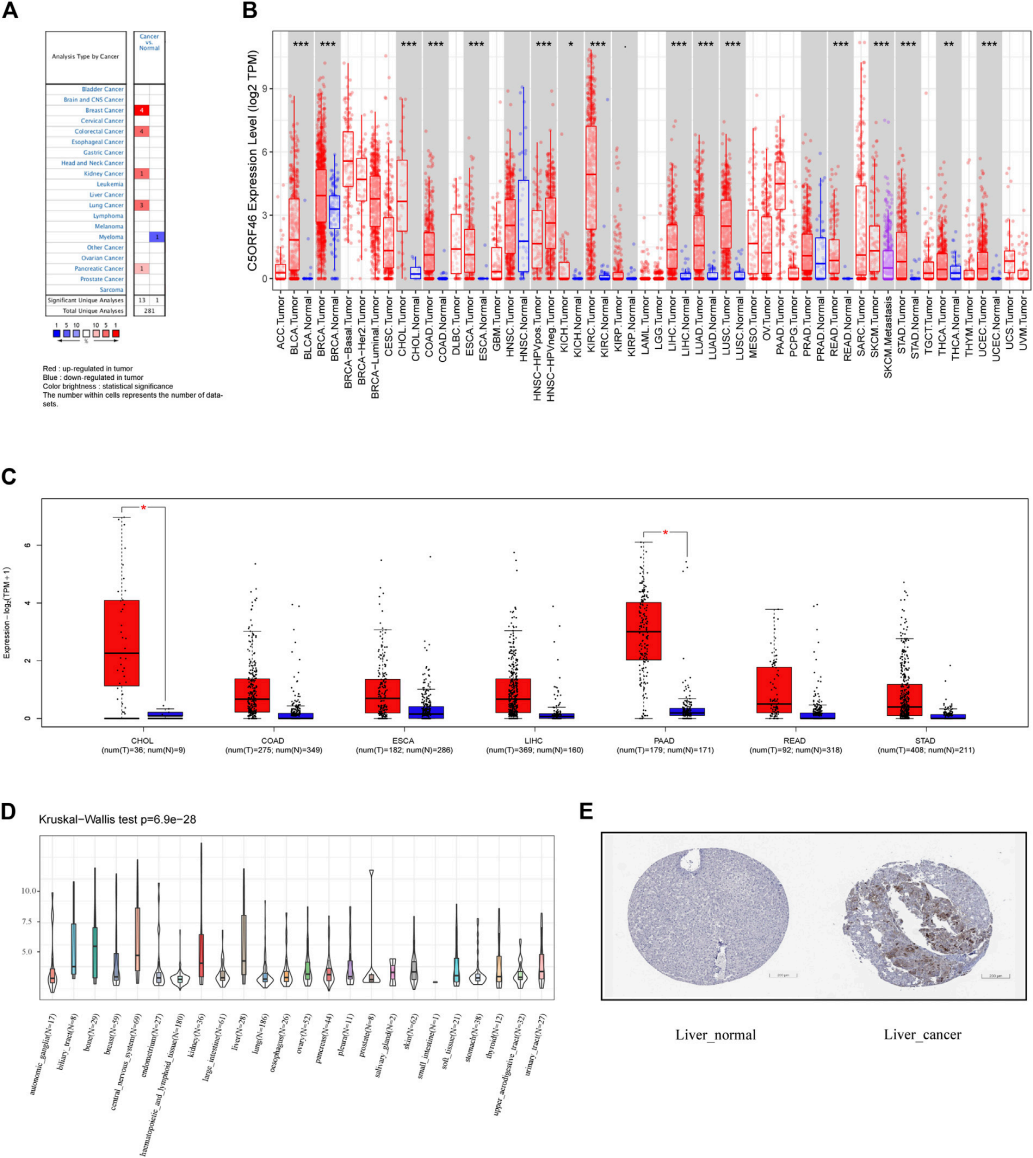
*C5ORF46* expression pattern in different types of cancers. **(A)** *C5ORF46* expression in different cancers and paired normal tissue in the Oncomine database (*p* value ≤ 1E-4, fold change ≥2, and gene rank ≥ top 10%). Red indicates upregulated expression and blue indicates downregulated expression in tumor. The color brightness is determined by statistical significance. The number within cells represents the number of datasets. **(B)** *C5ORF46* expression level in different tumors from TIMER database. (*p < 0.05, **p < 0.01, ***p < 0.001). **(C)** *C5ORF46* expression in seven GI tumors and paired normal tissue in the GEPIA database. (*p < 0.05, **p < 0.01, ***p < 0.001). **(D)** *C5ORF46* expression in tumor cell lines. **(E)** Immunohistochemistry images of *C5ORF46* gene expression in normal (left) and tumor (right) tissues analyzed by the Human Protein Atlas.

To build an understanding of the expression pattern of *C5ORF46* in different GI tumors, the Oncomine database was investigated. *C5ORF46* mRNA levels were found to be considerably increased in colorectal cancer and pancreatic cancer tissues compared to the corresponding normal tissues (Figure 1A). According to the TIMER database, the expression levels of *C5ORF46* were considerably higher in GI tumor tissues, namely CHOL, COAD, ESCA, LIHC, READ, and STAD. Because PAAD data were not available for its corresponding normal tissue, this cancer type was not compared. All significant *p*-value were <0.001 (Figure 1B). In supplementary results, TCGA and GTEx databases were integrated using R software to further analyze the expression pattern of *C5ORF46* in GI tumors (Supplementary Figure S1B). The findings from Spplementary Figure S1B consistently demonstrated that *C5ORF46* expression was considerably increased in six GI tumors: CHOL, COAD, ESCA, LIHC, READ, and STAD. In contrast to the Oncomine results, *C5ORF46* levels in PAAD were lower in tumor tissues than in tumor-adjacent normal tissues, although at insignificant levels. The boxplots generated from GEPIA showed that *C5ORF46* mRNA expression was significantly higher in CHOL and PAAD (Figure 1C). Relative *C5ORF46* expression levels in tumor cell lines were presented according to tissue origin across 24 tissue cell lines using the data from the CCLE database (Figure 1D). Of all the GI cell lines, *C5ORF46* expression was the highest in liver. As shown in Figure 1E, we downloaded a pair of representative IHC results from the HPA database to evaluate *C5ORF46* expression at the protein level. Liver demonstrated stronger *C5ORF46* staining in cancer tissues than in normal tissues. In terms of the cell line information, however, other GI tumors demonstrated no visible differences, possibly due to low baseline expression. Overall, the above evidence suggests that *C5ORF46* expression was elevated in GI tumor tissues.

### Analysis of *C5ORF46* genomic alterations

**FIGURE 2:**
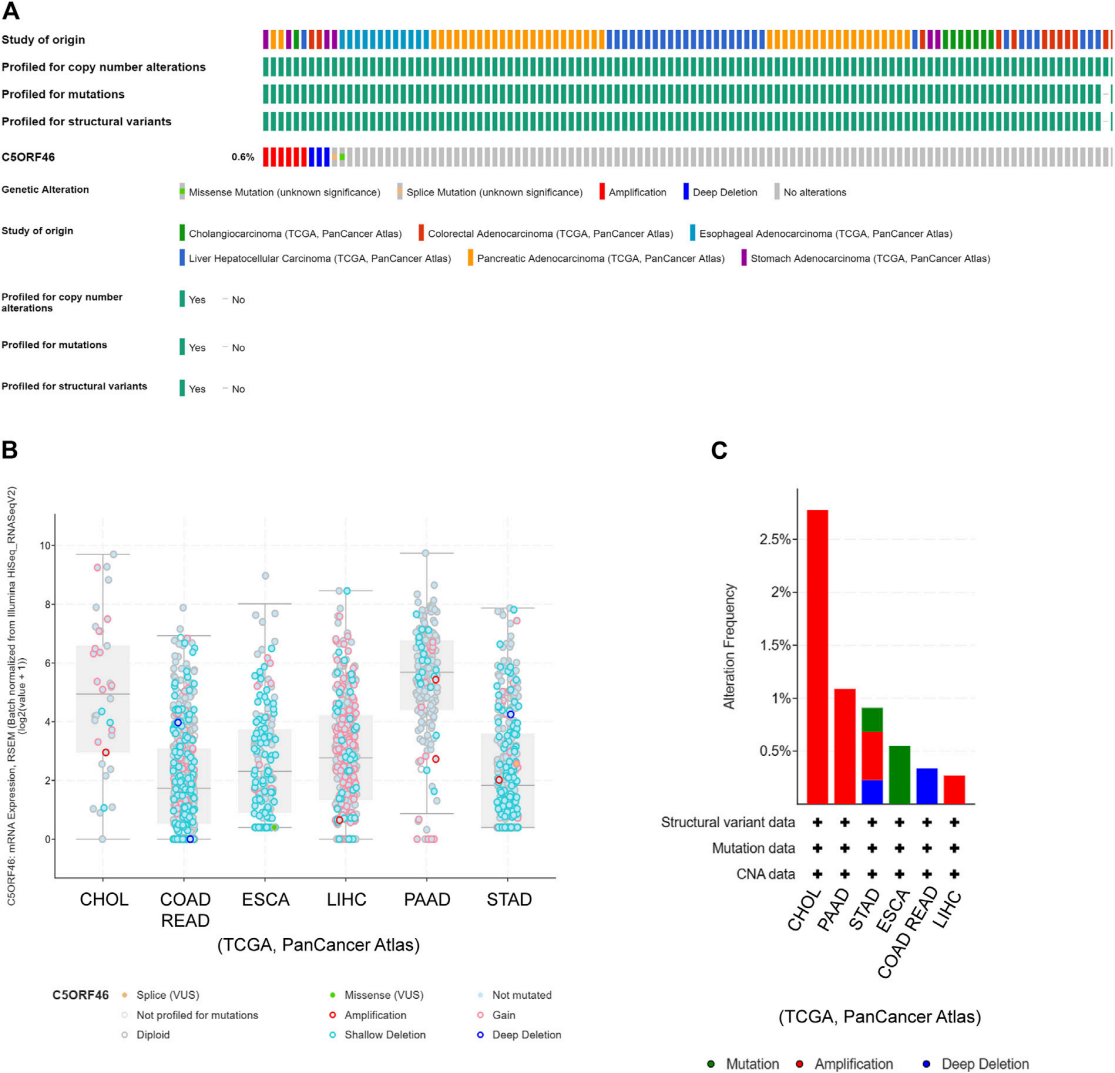
*C5ORF46* genomic alterations in GI tumors analyzed by the cBioPortal database. **(A)** OncoPrint shows genetic alterations of *C5ORF46* in GI tumors. **(B)** Mutation types of *C5ORF46* in GI tumors. **(C)** The alteration frequency of *C5ORF46* in GI tumors.

We used cBioPortal to investigate the relationship between *C5ORF46* mutation and tumor progression. The results indicate that genetic changes of *C5ORF46* were present in 0.6% of GI tumor patients based on data from TCGA, of which amplification was the most common type (Figure 2A). The mutational landscape indicated that *C5ORF46* alterations mainly consist of shallow deletion, gain, and diploid, resulting in gene expression alterations (Figure 2B). Copy number variation (CNV) mainly occurred in CHOL and PAAD, and amplification accounted for all of their copy number alterations (Figure 2C). These findings indicate that chromosomal changes of *C5ORF46* occur in cancer tissue and may play a role in cancer initiation and development.

### *C5ORF46* as a prognostic marker

**FIGURE 3:**
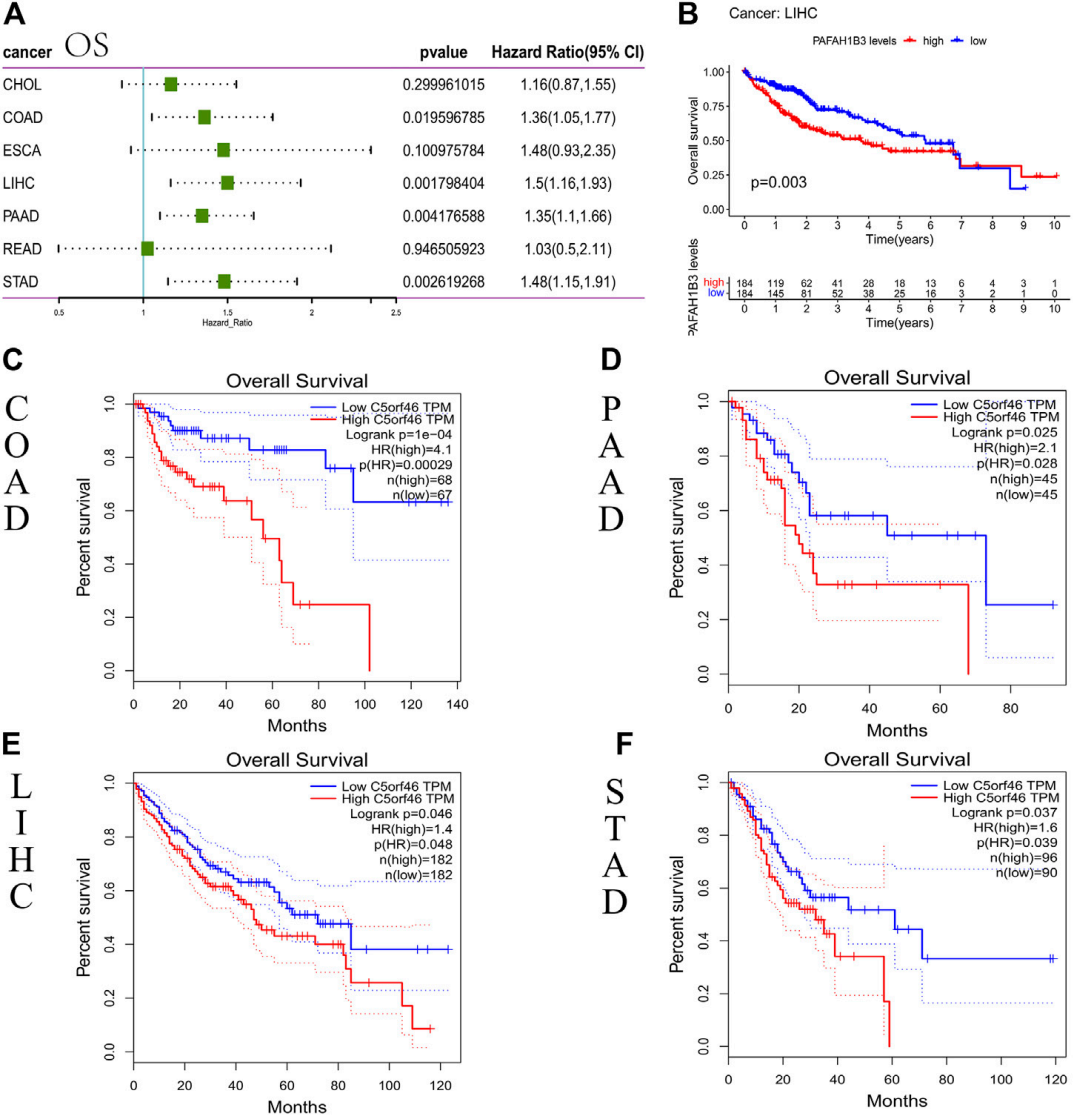
Association between *C5ORF46* expression and overall survival (OS) in GI tumors. **(A)** Forest plot of the association between *C5ORF46* expression and OS. **(B)** Kaplan-Meier analysis of the association between *C5ORF46* expression and OS. **(C–F)** Kaplan-Meier analysis of the association between *C5ORF46* expression and OS in GEPIA.

We carried out a survival association analysis for each GI tumor, including OS, DSS/DFS, and PFI, to investigate the relationship between *C5ORF46* expression level and prognosis in GI tumors. *C5ORF46* expression levels were linked with OS in COAD (HR = 1.36, *p* = 0.020), LIHC (HR = 1.5, *p* = 0.002), PAAD (HR = 1.35, *p* = 0.004), and STAD (HR = 1.48, *p* = 0.003), according to the Cox proportional hazards model study (Figure 3A). *C5ORF46* was a high-risk gene in all four tumors. High *C5ORF46* expression was similarly linked with poor OS in LIHC patients (Figure 3B; *p* = 0.026), according to Kaplan-Meier survival analysis. In GEPIA, we found, apart from LIHC (HR = 1.4, *p* = 0.048), that patients with higher *C5ORF46* expression had poorer OS could also be observed in COAD (HR = 4.1, *p* = 0.00029), PAAD (HR = 2.1, *p* = 0.028), and STAD (HR = 1.6, *p* = 0.039) by Kaplan-Meier survival analysis. These results were consistent with the forest plot results (Figures 3C–F). They suggest that high *C5ORF46* expression may be related to poor OS in COAD, LIHC, PAAD, and STAD.

**FIGURE 4:**
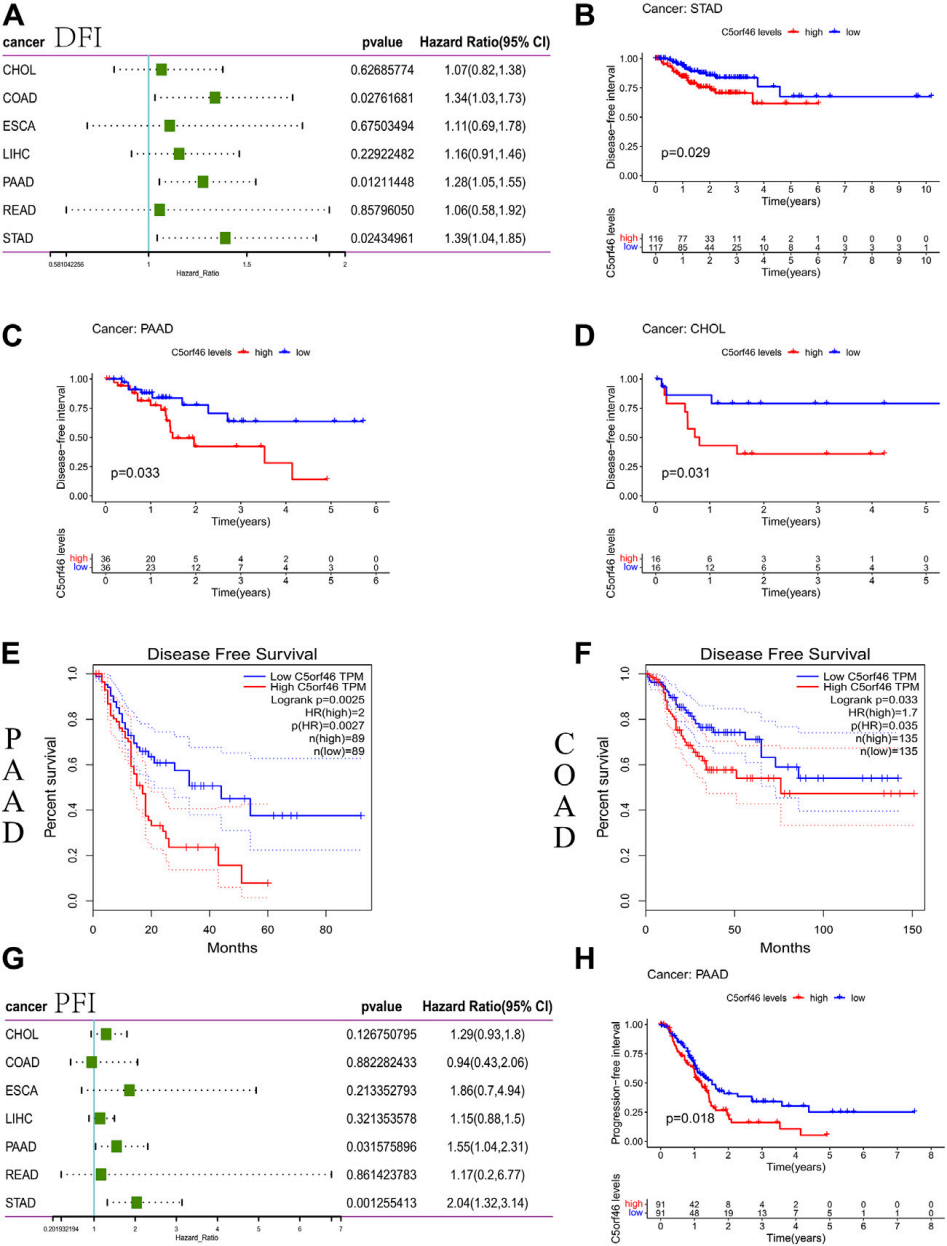
Association between *C5ORF46* expression and disease-free survival (DFS) or disease-free interval (DFI) and progression-free interval (PFI) in GI tumors. **(A)** Forest plot of the association between *C5ORF46* expression and DFS/DFI. **(B)** Kaplan-Meier analysis of the association between *C5ORF46* expression and PFI. **(C–F)** Kaplan-Meier analysis of the association between *C5ORF46* expression and PFI in GEPIA. **(G)** Forest plot of the association between *C5ORF46* expression and PFI. **(H)** Kaplan-Meier analysis of the association between *C5ORF46* expression and PFI.

The correlation between *C5ORF46* expression and DFS/DFI was evaluated with a cox proportional hazards model. The forest plots revealed significant associations between high *C5ORF46* expression and poor DFI in patients with COAD (HR = 1.34, *p* = 0.028), PAAD (HR = 1.28, *p* = 0.012), and STAD (HR = 1.39, *p* = 0.024) (Figure 4A). Kaplan-Meier survival analysis showed that *C5ORF46* expression levels correlated with poor DFI in patients with CHOL (*p* = 0.031), PAAD (*p* = 0.033), and STAD (*p* = 0.029; Figures 4B–D). In GEPIA, significant correlations were detected between *C5ORF46* and DSS in COAD (HR = 1.7, *p* = 0.035) and PAAD (HR = 2, *p* = 0.0027) by Kaplan-Meier survival analysis (Figures 4E,F). Thus, high *C5ORF46* expression may be related to poor DFS/DFI in CHOL, COAD, PAAD, and STAD.

Our forest plots revealed significant connections between high *C5ORF46* expression and low PFI in PAAD (HR = 1.55, *p* = 0.032) and STAD (HR = 2.04, *p* = 0.001) (Figure 4G). Kaplan-Meier analysis revealed that patients with high levels of *C5ORF46* expression had poor PFI in PAAD (*p* = 0.018; Figure 4H). Hence, high *C5ORF46* expression may be related to poor PFI in PAAD and STAD. These results suggest a negative correlation of *C5ORF46* expression and patient prognosis in GI tumors. Therefore, *C5ORF46* may be a possible prognostic indicator molecule in GI tumors.

### *C5ORF46* expression and clinical phenotypes

**FIGURE 5:**
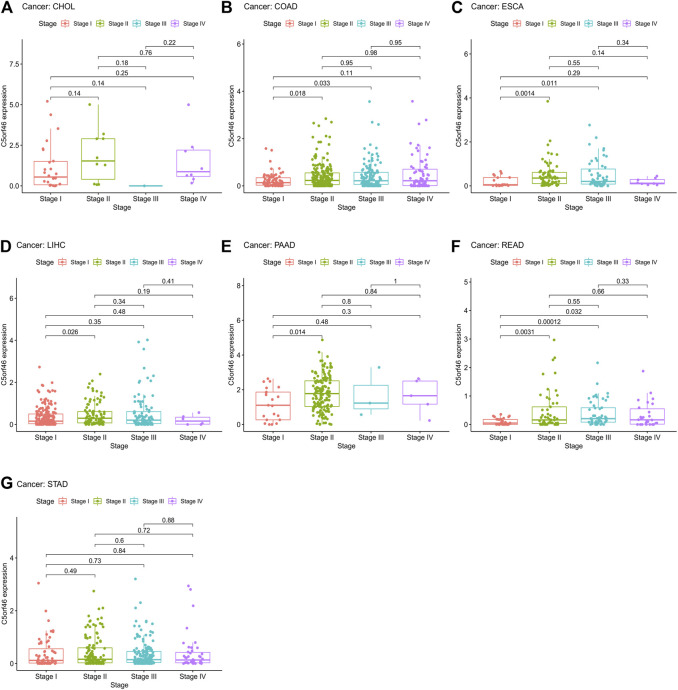
Association between *C5ORF46* expression and tumor stage in **(A)** cholangiocarcinoma (CHOL), **(B)** colon adenocarcinoma (COAD), **(C)** esophageal carcinoma (ESCA), **(D)** liver hepatocellular carcinoma (LIHC), **(E)** pancreatic adenocarcinoma (PAAD), **(F)** rectum adenocarcinoma (READ), **(G)** stomach adenocarcinoma (STAD).

Subsequently, differential analysis of *C5ORF46* expression profile was performed according to the staging of each tumor. We found *C5ORF46* expression displayed significant difference between stages I and II in five out of seven types of GI tumor, including COAD (*p* = 0.018; Figure 5B), ESCA (*p* = 0.0014; Figure 5C), LIHC (*p* = 0.026; Figure 5D), PAAD (*p* = 0.014; Figure 5E), and READ (*p* = 0.0031; Figure 5F). However, we found no significant *C5ORF46* expression differences between stages of CHOL (Figure 5A) and STAD (Figure 5G). Of the five GI tumors with statistical significance between *C5ORF46* expression and disease stage, in all cases, stage II presented a higher *C5ORF46* expression level compared with stage I. COAD (*p* = 0.033) and ESCA (*p* = 0.011) demonstrated differential *C5ORF46* expression between stages I and III. READ demonstrated additional differential *C5ORF46* expression between stage I and stage III (*p* = 0.00012) and stage IV (*p* = 0.032) respectively. In regard to COAD, ESCA, and READ, *C5ORF46* expression levels in stage I were the lowest. *C5ORF46* expression in READ patients underwent sequential upregulation through stage I, II, and III, that did not continue in stage IV.

We analyzed the relationship of *C5ORF46* expression and patient age. We discovered that patients ≤65 years had higher *C5ORF46* expression levels in the context of ESCA, while, in other GI tumors, no significant correlation was detected (Supplementary Figure S2).

### *C5ORF46* expression association with Tumor mutational burden and microsatellite instability

**FIGURE 6:**
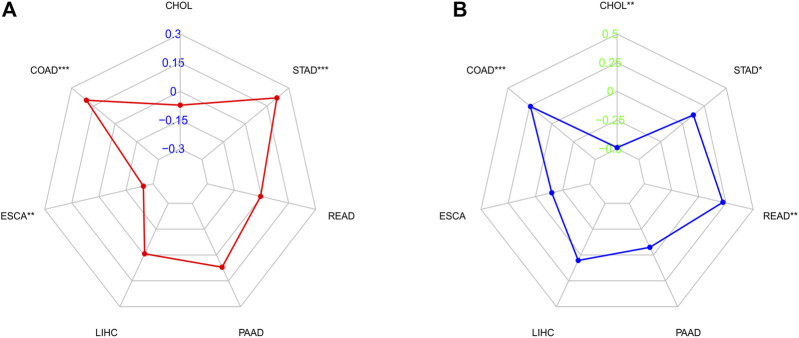
Correlation analysis between *C5ORF46* expression and tumor mutational burden (TMB), microsatellite instability (MSI) in GI tumors. **(A)** Results of correlation analysis between *C5ORF46* expression and TMB in GI tumors described using Spearman’s rank correlation coefficient. **(B)** Results of correlation analysis between *C5ORF46* expression and MSI in GI tumors described using Spearman’s rank correlation coefficient. (*p < 0.05, **p < 0.01, ***p < 0.001).

We investigated whether *C5ORF46* expression was linked to TMB and MSI. The total amount of nonsynonymous point mutations per coding region of a tumor genome is commonly referred to as TMB (Yarchoan et al., 2017; McNamara et al., 2020). MSI is a marker for defective DNA mismatch repair (dMMR). Cancers harboring a dMMR mechanism are often hypermutated, stacking alterations in monomorphic microsatellites that are prone to mismatch errors (Luchini et al., 2019). Together, MSI/dMMR and TMB can be used to determine which patients should receive immunotherapy, for example ICIs (Luchini et al., 2019). As shown in Figure 6A, using Spearman’s rank correlation coefficient, the correlation of *C5ORF46* expression with TMB was statistically examined for each GI tumor type independently. *C5ORF46* expression and TMB were shown to be positively linked in COAD and STAD, and negatively linked in ESCA. *C5ORF46* expression was evaluated for its correlation with MSI using Spearman’s rank correlation coefficient. As shown in Figure 6B, *C5ORF46* expression was significantly positively correlated with MSI in COAD, READ, and STAD, but negatively correlated with CHOL.

### Relationship between *C5ORF46* expression and tumor microenvironment

**FIGURE 7:**
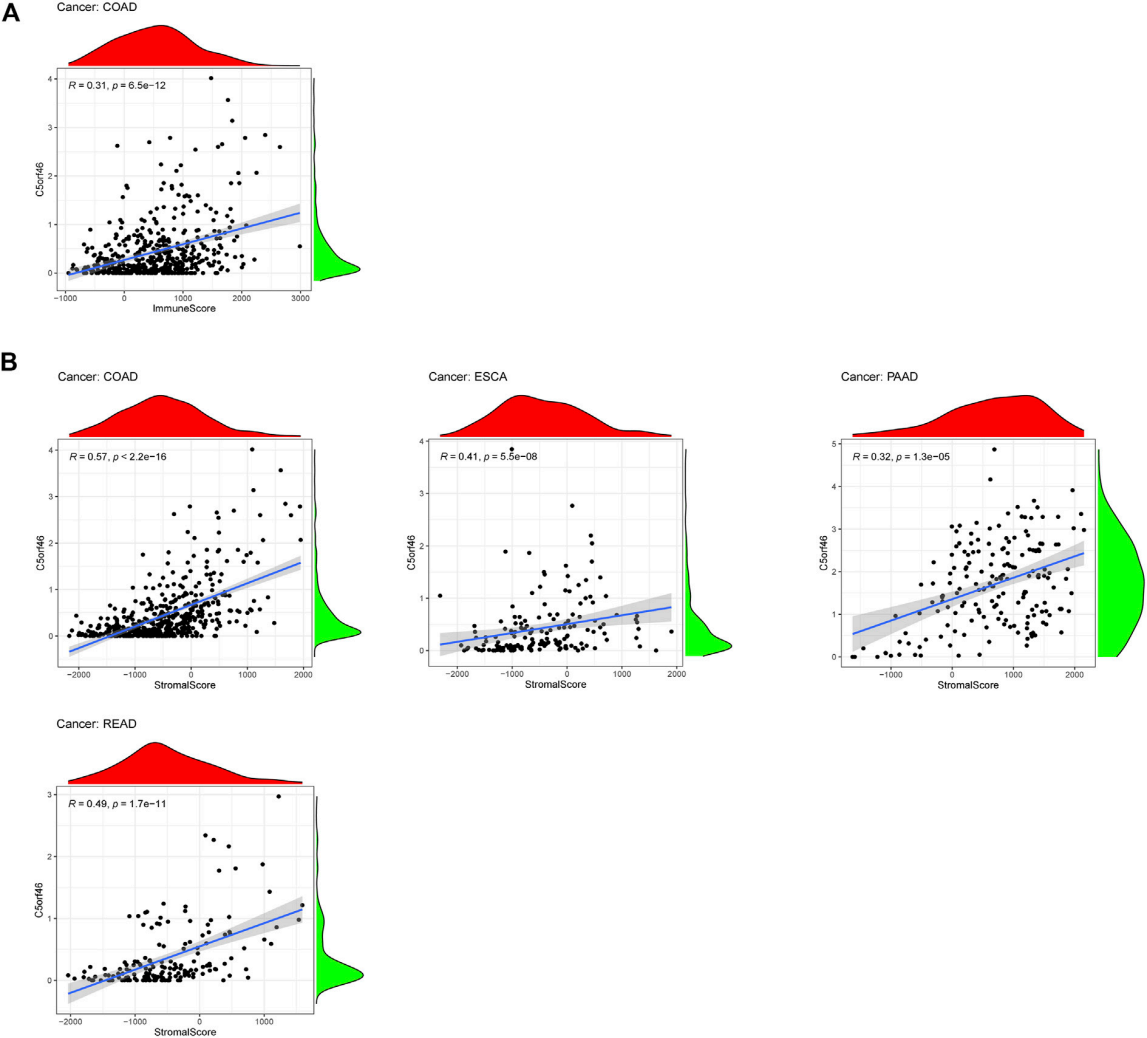
Correlation analysis between *C5ORF46* expression and tumor microenvironment in GI tumors. **(A)** Correlation analysis between *C5ORF46* expression and immune scores in colon adenocarcinoma (COAD). **(B)** Correlation analysis between *C5ORF46* and stromal scores in colon adenocarcinoma (COAD), esophageal carcinoma (ESCA), pancreatic adenocarcinoma (PAAD),and rectum adenocarcinoma (READ).

A growing body of evidence suggests that the tumor immunological microenvironment plays a critical role in tumor incidence and progression. Consequently, further research into the link between TME and *C5ORF46* expression in GI tumors is needed. In seven types of GI tumor, the ESTIMATE algorithm was used to produce stromal and immune cell scores, and associations between *C5ORF46* expression levels and these two scores were investigated. *C5ORF46* expression was found to be significantly and positively linked to immunological ratings in COAD (R = 0.31, *p* = 6.5e^−12^; Figure 7A). However, for most of the GI tumors, the correlations of *C5ORF46* expression and immunological ratings were weak (Supplementary Figure S3) (Akoglu, 2018). For *C5ORF46* expression and stromal scores, positive correlations were observed in COAD (R = 0.57, *p* = 2.2 E^−16^), ESCA (R = 0.41, *p* = 5.5e^−8^), PAAD (R = 0.32, *p* = 1.3e^−5^), and READ (R = 0.49, *p* = 1.7e^−11^; Figure 7B). The insignificant results for other GI tumors are shown in Supplemenatry Figure S3. In the context of COAD, the correlation coefficients of *C5ORF46* expression with both immunological compartment (R = 0.31) and stromal compartment (R = 0.57) were relatively high for other GI tumors, both of which could be defined as “fair” (correlation coefficient value: 0.30–0.50) with high statistical significance according to Chan (2003) (*p* < 0.0001) (Chan, 2003).

### Relationship between *C5ORF46* expression and tumor immune cell infiltration levels

We examined the relationship between *C5ORF46* expression and the levels of infiltration of 22 immune-related cells in GI tumors using CIBERSORT. Our data indicated that 1) *C5ORF46* expression levels were significantly and positively associated with neutrophils in CHOL (R = 0.59, *p* = 0.00034) and PAAD (R = 0.34, *p* = 6.7e^−6^), with dendritic cells resting in CHOL (R = 0.48, *p* = 0.0054), and with macrophages M2 in READ (R = 0.40, *p* = 2.4e^−7^); and 2) *C5ORF46* expression levels were significantly and negatively associated with both B cells naïve (R = -0.32, *p* = 8.9e^−5^) and T cells regulatory (R = −0.34, *p* = 1.8e^−5^) in ESCA (Table 2).

**TABLE 2:** Relationship between *C5ORF46* expression and immune cell infiltration in GI tumors. (*: |R|≥0.3 and *p* < 0.05, **: |R|≥0.3 and *p* < 0.01, ***: |R|≥0.3 and *p* < 0.001).

Cell type	CHOL (*p*-value/Cor)	COAD (*p*-value/Cor)	ESCA (*p*-value/Cor)	LIHC (*p*-value/Cor)	PAAD (*p*-value/Cor)	READ (*p*-value/Cor)	STAD (*p*-value/Cor)
B cells memory	0.69/0.073	0.35/−0.044	0.093/0.14	0	0.12/0.12	0.26/−0.092	0.65/−0.024
B cells naive	0.82/−0.041	0.65/0.022	8.9e^−05***^/−0.32	0.83/0.013	0.093/−0.13	0.39/−0.07	1.2e^−07^/−0.27
Dendritic cells activated	0.071/−0.32	0.065/−0.088	0.16/0.12	0.074/0.1	0.17/0.11	0.072/−0.15	0.8/0.014
Dendritic cells resting	0.0054^**^/0.48	0.022/−0.11	0.054/0.16	0.48/0.041	0.92/−0.0078	0.26/0.091	0.027/−0.11
Eosinophils	0	0.92/0.0048	0	0	0	0.69/0.033	0.47/0.037
Macrophages M0	0.32/0.18	0.00019/0.18	0.094/0.14	1e^−05^/0.25	0.00015/0.29	0.022/0.19	8.5e^−06^/0.23
Macrophages M1	0.91/-0.02	4.5e^−05^/0.19	0.098/0.14	0.84/0.012	0.44/0.059	0.038^*^/0.17	8.9e^−06^/0.23
Macrophages M2	0.89/−0.026	7.9e^−10^/0.29	0.23/0.1	0.011/−0.15	0.6/0.04	2.4e^−07***^/0.4	6e^−05^/0.21
Mast cells activated	0.22/0.22	0.0068/0.13	0.34/0.079	0	0	0.32/0.081	0.015/0.13
Mast cells resting	0.26/-0.2	0.036/−0.1	0.48/−0.058	0.25/0.067	0.043^*^/−0.16	0.048/−0.16	0.0017/−0.16
Monocytes	0.24/-0.21	0.16/0.067	0.099/0.14	0.12/−0.091	0.095/−0.13	0.43/0.065	0.6/−0.028
Neutrophils	0.00034^***^/0.59	2.7e^−07^/0.24	0.69/−0.033	0.0023/0.18	6.7e^−06***^/0.34	0.17/0.11	6.2e^−05^/0.21
NK cells activated	0.19/−0.24	0.64/0.022	0.11/0.13	0.25/0.068	0.049/0.15	0.21/−0.1	0.0072/0.14
NK cells resting	0	0.4/0.041	0.17/0.11	0.063/−0.11	0.48/−0.054	0.82/−0.019	0.04/0.11
Plasma cells	0.72/0.066	0.00017/-0.18	0.075/−0.15	0.29/0.062	0.98/−0.0021	0.091/−0.14	0.18/−0.069
T cells CD4 naive	0	0	0	0	0	0	0
T cells CD4 memory activated	0.7/0.07	2.2e^−05^/-0.2	0.96/0.0043	0.35/0.054	0.76/−0.024	0.36/−0.074	0.018/0.12
T cells CD4 memory resting	0.43/0.14	0.001/−0.16	0.16/−0.12	0.017/−0.14	0.62/0.038	0.033/−0.17	2e^−04^/−0.19
T cells CD8	0.077/−0.32	0.98/−0.001	0.56/−0.048	0.52/−0.037	0.028/−0.17	0.62/0.041	0.96/−0.0024
T cells follicular helper	0.22/−0.22	0.22/−0.058	0.3/0.086	0.034/0.12	0.88/0.012	0.56/−0.048	0.073/0.093
T cells regulatory (Tregs)	0.079/0.31	0.54/0.029	1.8e^−05***^/-0.34	0.96/0.0032	0.7/0.03	0.48/0.058	0.12/−0.081
T cells gamma delta	0	0	0	0.16/−0.082	0	0	0

**FIGURE 8:**
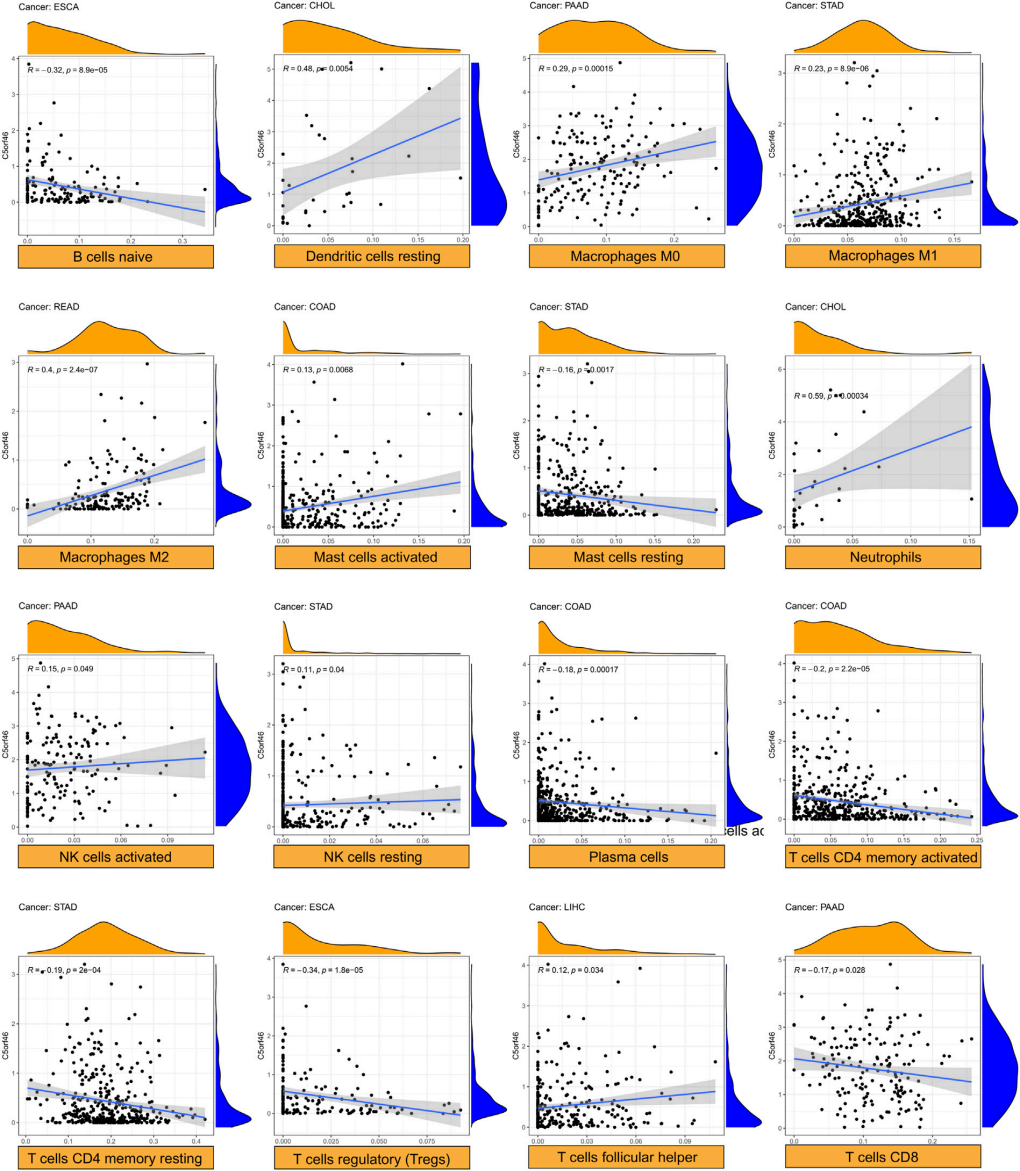
Relationship between *C5ORF46* expression and tumor infiltration of different immune cells. (Tumors with the highest correlation coefficients in each type of immune cells).

For each kind of immune cell, Figure 8 shows the tumors with the strongest correlation coefficients between degree of infiltration and *C5ORF46* expression. Other significant results are visualized in Supplementary Figure S4. Here, *C5ORF46* expression had the highest correlation coefficient with neutrophils in CHOL (R = 0.59, *p* = 0.00034).

**FIGURE 9:**
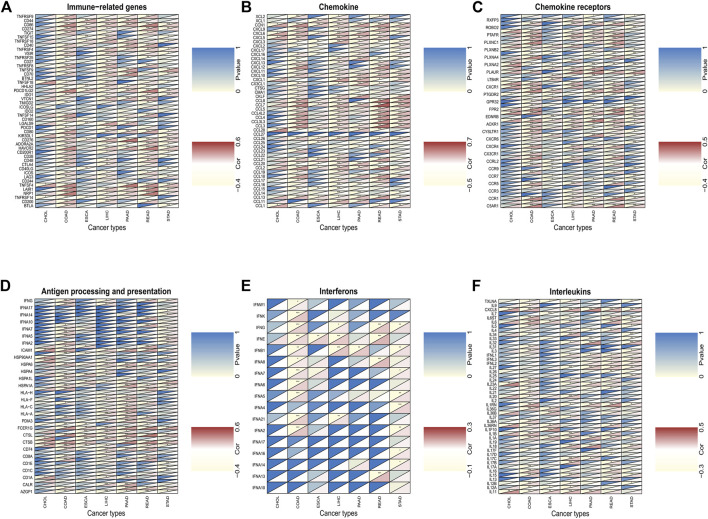
The association between *C5ORF46* and **(A)** immune checkpoint genes; **(B)** chemokine-related genes; **(C)** chemokine receptors-related genes; **(D)** antigen processing and presentation-related genes; **(E)** interferons-related genes; **(F)** interleukins-related genes (*p < 0.05, **p < 0.01, ***p < 0.001).

We investigated the associations between *C5ORF46* expression and immune checkpoint genes in seven GI tumors (Figure 9A). We also analyzed specific immune-modulating genes encoding chemokine (Figure 9B), chemokine receptors (Figure 9C), antigen processing and presentation proteins (Figure 9D), interferons (Figure 9E), and interleukins (Figure 9F). As shown in Figure 9A, *C5ORF46* expression demonstrated significant and positive correlations with immune checkpoint genes, for example, PD-L1 (CD274) in COAD (R = 0.34, *p* = 5.59e^−14^) and READ (R = 0.35, *p* = 3.74e^−06^). Figures 9A,B demonstrate that C5ORF46 expression significantly and positively correlated with genes encoding chemokine and chemokine receptors, especially in COAD and READ. The results show that the majority of significant correlations between *C5ORF46* expression and immune-related genes in GI tumors were positive, suggesting that *C5ORF46* may play a role in modulating the pattern of tumor immunity by regulating the expression levels of the above-mentioned immune-related genes.

### Kyoto encyclopedia of genes and genomes pathway enrichment analysis and gene set enrichment analysis

**FIGURE 10:**
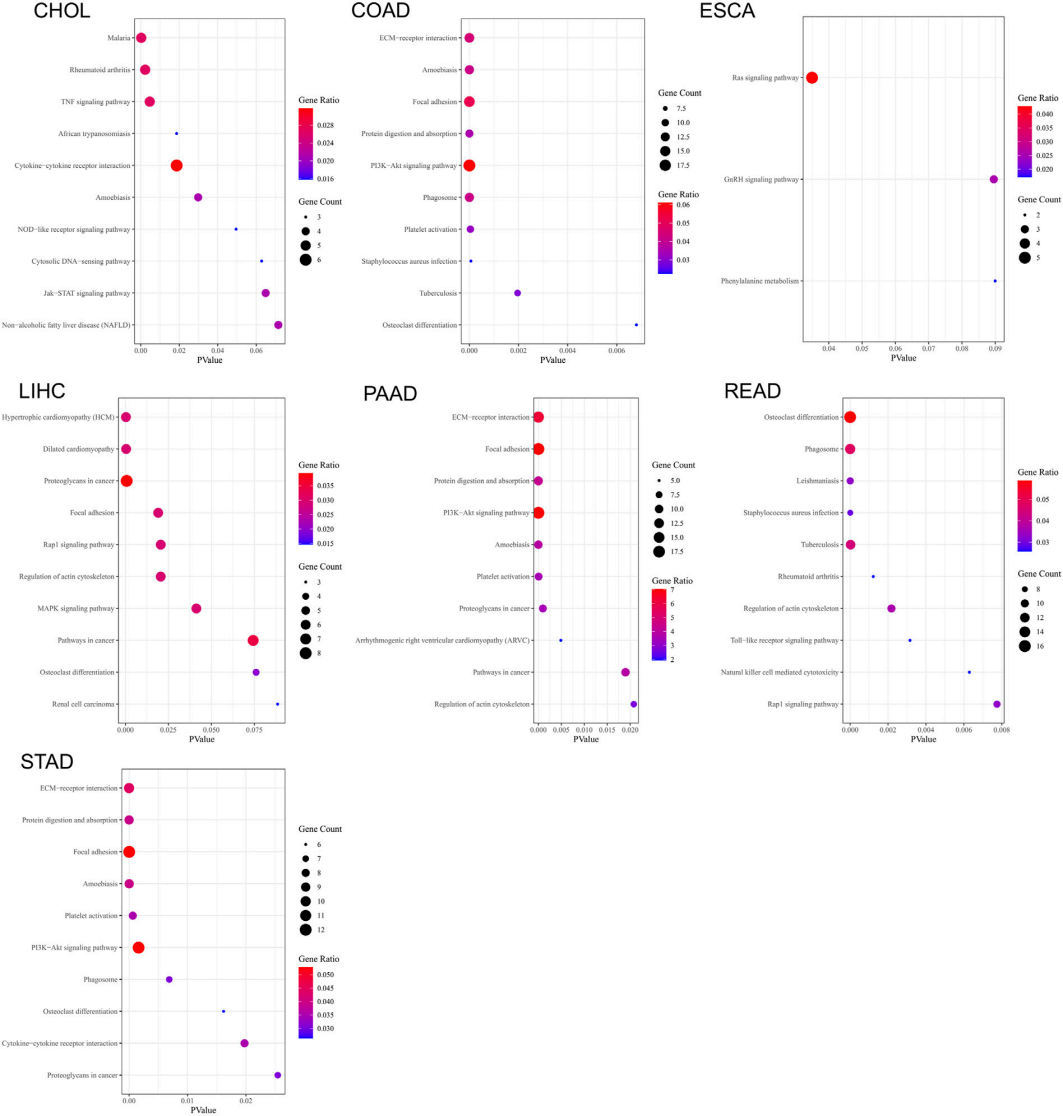
Bubble charts of kyoto encyclopedia of genes and genomes (KEGG) pathway enrichment analysis based on the top 300 genes that display similar expression patterns to *C5ORF46* in seven GI tumors.

We performed KEGG pathway enrichment analysis on the genes that display similar expression patterns to *C5ORF46* in GI tumors (Figure 10). The results show that these genes were significantly (*p* < 0.05) enriched in ameobiasis in CHOL, COAD, PAAD, and STAD, in focal adhesion in COAD, LIHC, PAAD, and STAD, in phagosome in COAD, READ, and STAD, in PI3K-Akt signaling pathway, protein digestion and absorption, platelet activation in COAD, PAAD, and STAD, in proteoglycans in cancer in LIHC, PAAD, and STAD, in regulation of actin cytoskeletion in LIHC, PAAD, and READ, in Rap1 signaling pathway in COAD, LIHC, and READ, in osteoclast differentiation in COAD and STAD, in staphylococcus aureus infection and tuberculosis in COAD and READ, in cytokine-cytokine receptor interaction in CHOL and STAD, and in rheumatoid arthritis in CHOL and READ.

**FIGURE 11:**
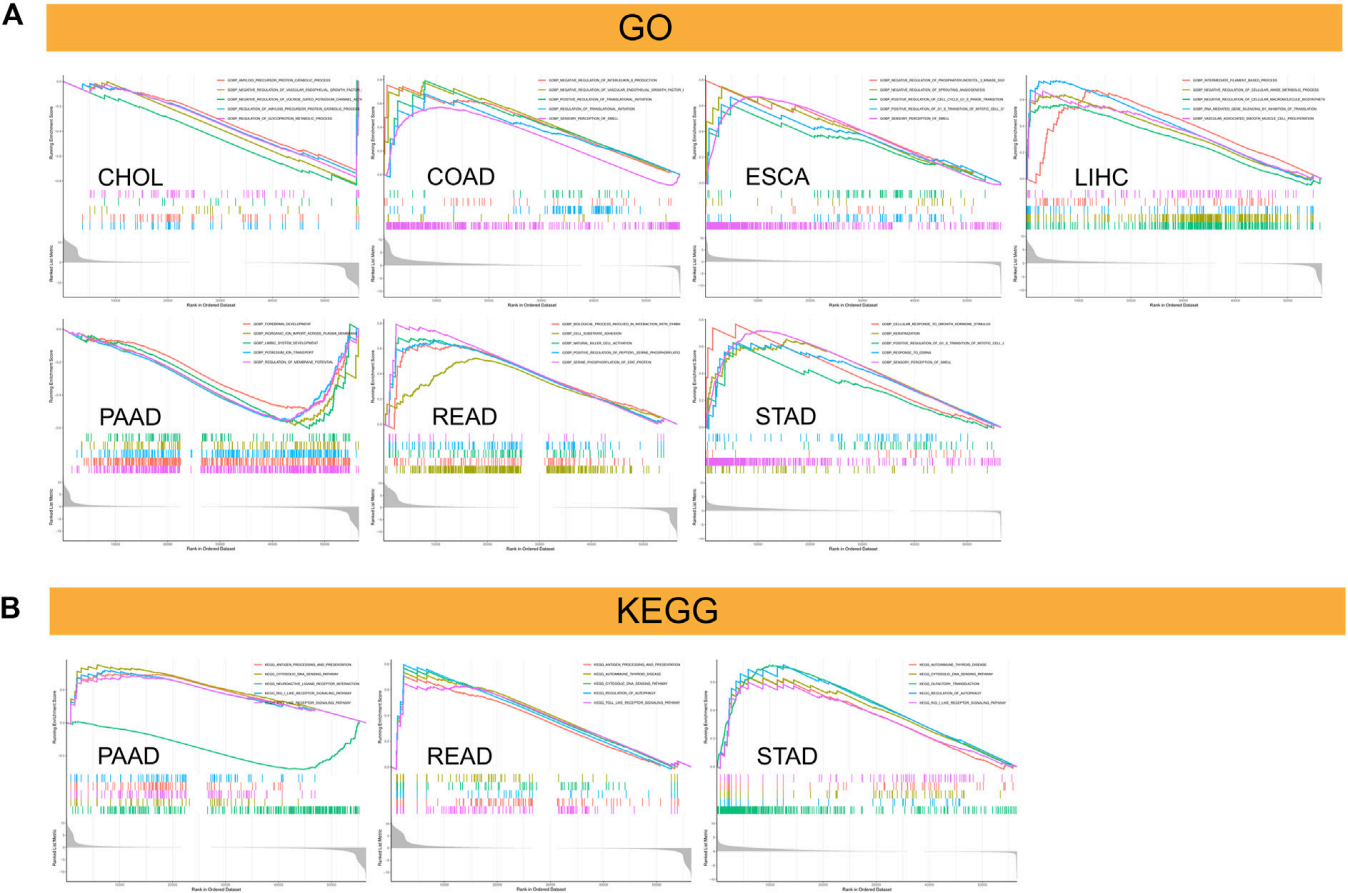
Results of Gene set enrichment analysis (GSEA). **(A)** Gene Ontology (GO) biological processes (BP) functional annotation of *C5ORF46* in GI tumors. **(B)** KEGG pathway analysis of *C5ORF46* in multiple cancers. Curves of different colors show different functions or pathways regulated in different cancers. Peaks on the upward curve indicate positive regulation and peaks on the downward curve indicate negative regulation.

We separated the TCGA tumor samples into two groups with high and low *C5ORF46* expression levels and analyzed the main terms of GO BP and KEGG pathway by GSEA to examine the influence of *C5ORF46* gene expression on GI tumors. The results of GO BP annotation showed that *C5ORF46* positively regulates cell cycle in ESCA and STAD. As for immune-related functions, *C5ORF46* was found to positively regulate NK cell activation in READ, yet negatively regulates interleukin 8 production in COAD. *C5ORF46* negatively regulates several metabolic processes in CHOL and LIHC. Contrasting results were found for regulation of VEGF production and angiogenesis in CHOL, COAD and ESCA tumor cells (Figure 11A). The KEGG terms indicated that *C5ORF46* activates several immune-related pathways in PAAD, READ and STAD, including antigen processing and presentation, cytosolic DNA-sensing signaling, regulation of autophagy, autoimmune thyroid disease pathways, and RIG-I-like receptor signaling and Toll-like receptor signaling. These play essential roles in innate antiviral immunity (Figure 11B) (Kawai and Akira, 2008; Onomoto et al., 2021). Genes enriched in the high C5ORF46 expression group were mainly from the interferon alpha (IFNA) family, for example IFNA4, IFNA8, IFNA6, and IFNA14, the major effector cytokines in mediating a host immune responses against viral infections (González-Navajas et al., 2012). Together, these results suggest that *C5ORF46* expression might play an essential role in human GI tumors by regulating inflammation and immune responses.

### Expression of *C5ORF46* in clinical samples

**FIGURE 12:**
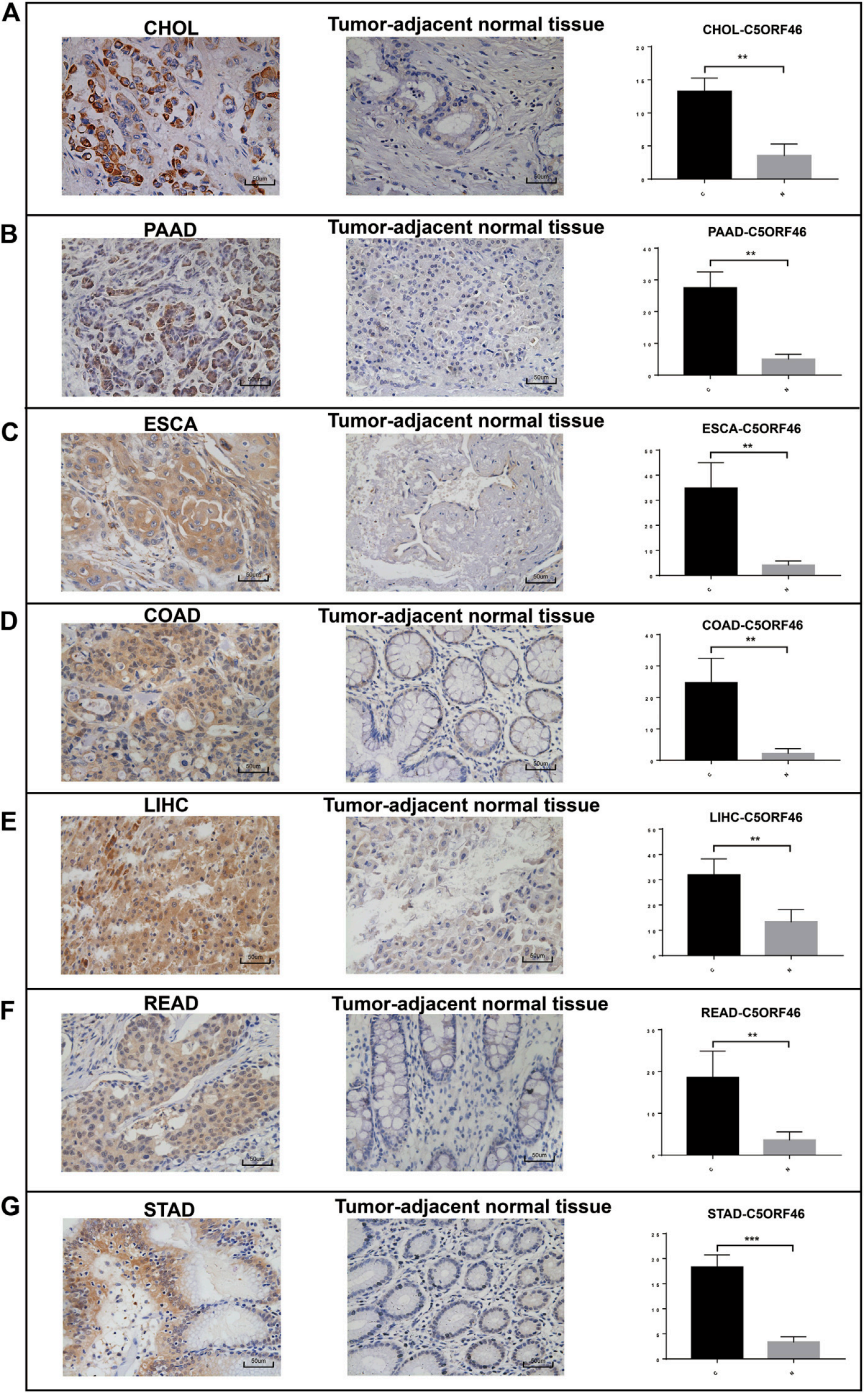
Upregulated *C5ORF46* expression in GI tumors validated by clinical samples. Representative images and quantification of IHC staining for *C5ORF46* in **(A)** CHOL, **(B)** PAAD, **(C)** ESCA, **(D)** COAD, **(E)** LIHC, **(F)** READ, and **(G)** STAD. Scale bars: 50 μm. *p*-values were obtained by unpaired *t*-test. All data are represented by mean ± SD. (C: tumor tissue; N: tumor-adjacent normal tissue).

To further verify our bioinformatics results, we evaluated *C5ORF46* expression in clinical GI tumor and tumor-adjacent tissues using IHC (Figure 12). The results show that *C5ORF46* was significantly upregulated in all the GI tumors compared to tumor-adjacent tissues (*p* < 0.05).

## Discussion

*C5ORF46*, also known as *SSSP1* and *AP-64*, is a protein-encoding gene located on chromosome 5q32. Although the function of *C5ORF46* remains unknown, it has been implicated in several diseases in terms of anti-inflammatory activities. Vesicular hand eczem (VHE) can be defined as a type of dermatitis that clinically manifests as vesicles that frequently present on the palms, palmar, or lateral portions of the digits (Menné et al., 2011; Agner et al., 2015). RNA-seq results revealed that *C5ORF46* is the most downregulated gene in lesioned VHE skin (Voorberg et al., 2021). Further, *C5ORF46* has been shown to encode an antimicrobial peptide *AP-64*, and exhibit antimicrobial activity against gram-negative bacteria (Zhong et al., 2021). *C5ORF46* is thought to interact with *TMBIM6*, a protein that regulates calcium homeostasis in the endoplasmic reticulum (Voorberg et al., 2021; BioGRID, 2022). Given that endoplasmic reticulum stress and calcium dysregulation are closely related to inflammatory responses and cancer immunity (So, 2018; Zhou et al., 2020), we postulated that *C5ORF46* may play a critical role in cancer inception and development. In support, several other studies have referred to the role of *C5ORF46* in carcinogenesis. By analyzing the RNA-seq data and clinical information of 169 recurrent glioblastoma (GBM) samples obtained from TCGA, Tang et al. (2021) identified *C5ORF46* as one of the DEG signatures that may account for the recurrent status of GBM. They found *C5ORF46* was related to poor prognosis in recurrent GBM (rGBM) patients (Tang et al., 2021). Similarly, by analyzing the TCGA expression profile, *C5ORF46* was found to be differentially expressed and have prognostic significance in the context of stomach cancer (Cheng et al., 2020) and colorectal cancer (Chen et al., 2021). Further, the potential of exosomal *C5ORF46* as a tumor marker in pancreatic cancer was uncovered by bioinformatics analyses using multiple ORF databases (Makler and Narayanan, 2017). However, Makler and Narayanan’s results also implied that *C5ORF46* has restricted expression in the pancreas, as is the case in the whole gastrointestinal tract.

In this study, we first analyzed *C5ORF46* expression between tumor and tumor-adjacent normal tissues. Our results show that the expression of *C5ORF46* was significantly upregulated in all seven GI tumor tissues compared with normal tissues. In concert, our IHC results performed on clinical sections show increased *C5ORF46* protein levels in all the seven GI tumors compared to tumor-adjacent normal tissues. Based on available evidence in relation to the entire GI tract, *C5ORF46* demonstrated the highest expression levels in liver tissues and liver cell lines. It is possible, therefore, that *C5ORF46* may be a predictive marker for GI tumors.

Our study investigated the impact of *C5ORF46* on genomic alterations. As a biomarker of disease, changes, including deletion and amplification, at the chromosome level have become the focus of disease-related research in which CNV is an important pro-tumoral mechanism. Although the types of *C5ORF46* alteration were diverse and differed across cancers, in patients with CHOL and PAAD, who had the highest frequency of *C5ORF46* CNV alteration, all the alterations were amplification. These findings suggest that DNA copy number amplification in PAAD and CHOL may result in aberrantly activated *C5ORF46* gene expression, which could increase the probability of GI tumor formation.

We examined the prognostic values of *C5ORF46* by COX regression and Kaplan-Meier analysis using both the data downloaded from TCGA and from the GEPIA website. In concert with previous studies, high *C5ORF46* expression correlated significantly with poor prognosis in GI tumor patients. COX regression and Kaplan-Meier curves suggested that high *C5ORF46* expression is associated with: 1) poor OS in COAD, PAAD, LIHC and STAD; 2) poor DFI/DFS in CHOL, COAD, PAAD and STAD; and 3) poor PFI in PAAD. These results are consistent with the above-mentioned bioinformatics studies which implicate *C5ORF46* as a prognostic marker in COAD, PAAD and STAD.

We discovered that *C5ORF46* expression is related to tumor stage in COAD, ESCA, LIHC, PAAD, and READ. The expression levels of *C5ORF46* were significantly higher in stage II than in stage I. Intriguingly, most of the GI tumors tended to have the lowest expression levels of *C5ORF46* in stage I. We found that *C5ORF46* expression was particularly higher in younger patients (age ≤65) in ESCA. These findings show that *C5ORF46* expression levels may have the potential to guide the choice of treatment for GI tumor patients at different stages and ages.

TMB and MSI are viable prognostic markers for GI tumors. They have been reported to predict the efficacy of immunotherapy (Xiao and Freeman, 2015; Lee et al., 2019). We found that *C5ORF46* positively correlated with TMB and MSI in COAD and STAD, suggesting that high *C5ORF46* expression may serve as a marker for immune checkpoint blockade therapy sensitivity in COAD and STAD patients. Further, according to the ESTIMATE results, *C5ORF46* was positively related with immune and stromal scores in GI tumors, especially COAD, which indicated an increased ratio of corresponding components in the TME. These results suggest that, although *C5ORF46* is connected with poor prognosis in GI tumors, it positively regulates the immune cell infiltration, turning the tumor from “cold” to “hot,” thus marking a *C5ORF46*-high subgroup able to be targeted for immunotherapy.

To further study the relationship of *C5ORF46* and cancer immunity, we focused on the relationship between *C5ORF46* expression and the infiltration of 22 types of immune-related cells. Our study demonstrated that, in GI tumors, *C5ORF46* had a strong association with tumor-infiltrating immune cells, particularly neutrophils. Neutrophils account for about 60% of all leukocytes in circulation, and their antibacterial functions have been mostly studied in the context of inflammation and bleeding (Kim and Bae, 2016). However, after their infiltration into the TME, neutrophils become immunosuppressive tumor-associated neutrophils (TANs) which support tumor progression (Nagaraj et al., 2010; Brennecke et al., 2015). Additionally, the degree of TAN infiltration has been closely related to tumor stage and more aggressive phenotypes (Fossati et al., 1999; Reid et al., 2011). This gave a hint of how *C5ORF46* could participate in maintaining the TME and the progression of GI tumors. We found a strong relationship between *C5ORF46* expression and an increased expression of genes encoding immune checkpoint proteins, chemokine, chemokine receptors, antigen processing and presentation proteins, and interleukins, especially in COAD and READ patients. These findings provide a theoretical basis for combining molecular targeting and immunotherapy. Further, GSEA and KEGG pathway enrichment analysis indicated that *C5ORF46* could possess several immune-related functions and could play a role in regulating host inflammation and immune responses. These results strongly suggest the potential of *C5ORF46* as a target for cancer immunotherapy.

The role of aberrant *C5ORF46* expression in cancer development, patient prognosis, and cancer immunity was revealed in this study, and warrants further exploration. Since our study was based on bioinformatics and relied on public databases, bias may have occurred. Firstly, depending on the source, the data collection and process used to create the data could be inconsistent. This could influence the findings from some of our analyses. Secondly, neither the results nor the conclusions have been confirmed experimentally or prospectively in the laboratory or in the clinic. *In vivo* and *in vitro* studies are needed to do so. Thirdly, although we observed positive correlations of *C5ORF46* and neutrophils in the TME, in recent years two distinct subtypes of TANs have been suggested: a pro-tumorigenic (N2) phenotype and an antitumorigenic (N1) phenotype (Fridlender and Albelda, 2012). We did not describe or discuss this dichotomy of TANs, which requires further exploration. Fourthly, despite *C5ORF46* expression being shown to be substantially linked to immune cell infiltration and cancer prognosis in humans, there is no direct evidence that *C5ORF46* influences prognosis by participating in immune infiltration. Hence, the mechanisms by which *C5ORF46* participates in cancer progression and immune regulation remain to be clarified.

## Data Availability

The datasets presented in this study can be found in online repositories. The names of the repository/repositories and accession number(s) can be found below: https://www.ncbi.nlm.nih.gov/geo/query/acc.cgi?acc=GSE200427.
