# Supplementary figures and images for: A systematic analysis of C5ORF46 in gastrointestinal tumors as a potential prognostic and immunological biomarker
Source: Front Genet. 2022 Aug 5;13:926943. doi: 10.3389/fgene.2022.926943 (PMC9389054; doi:10.3389/fgene.2022.926943)

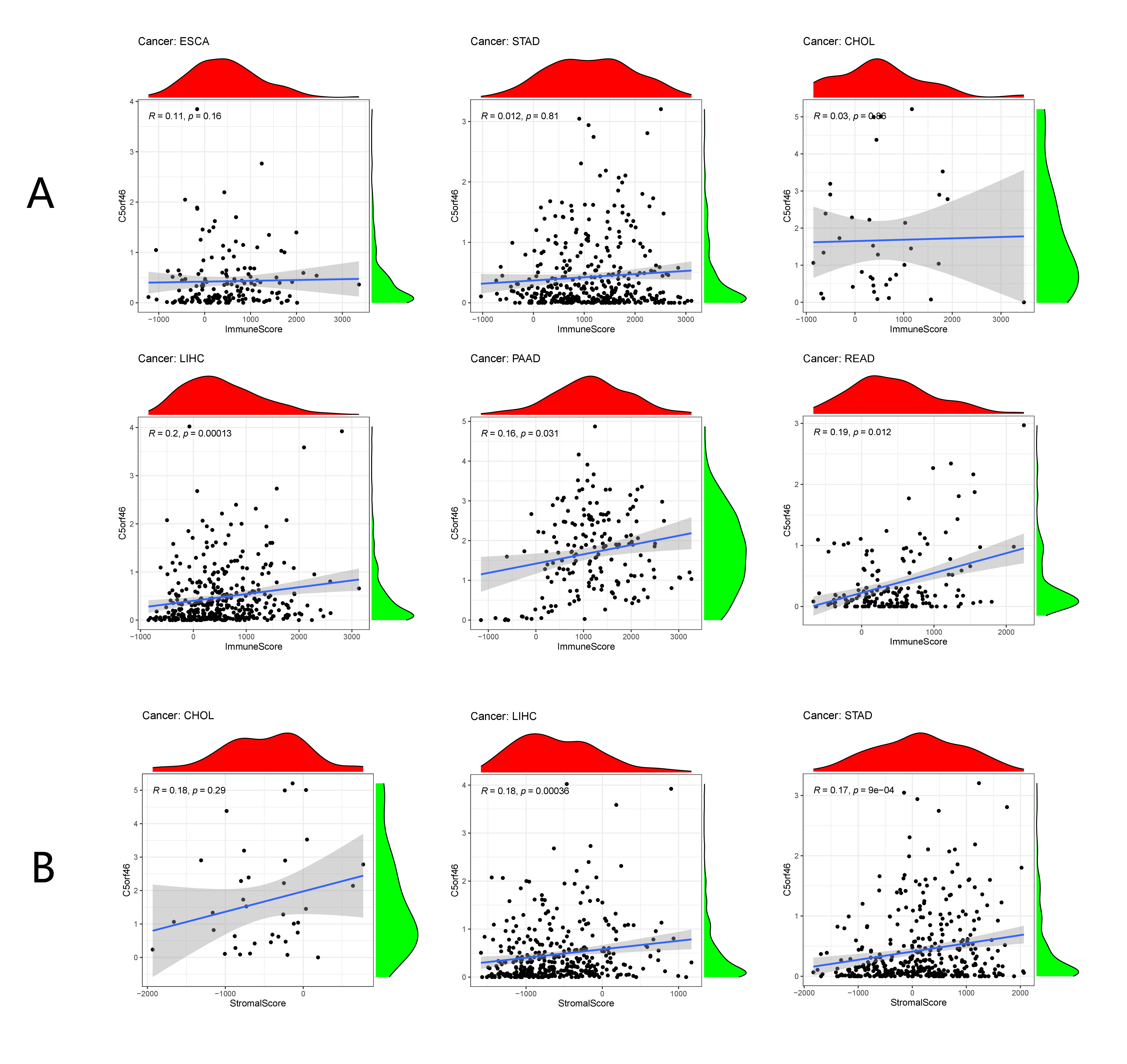

Supplement: Supplementary file 1 [file Image3.JPEG]

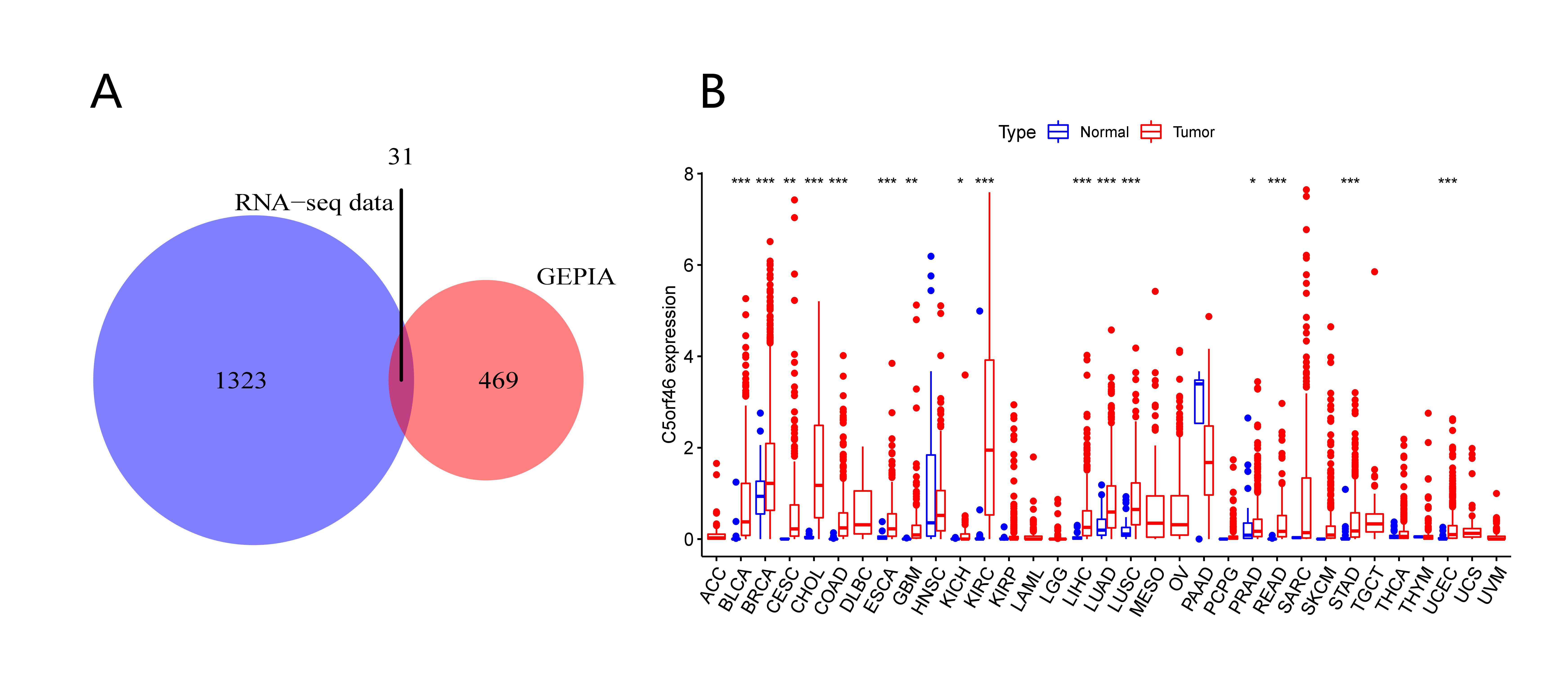

Supplement: Supplementary file 2 [file Image1.JPEG]

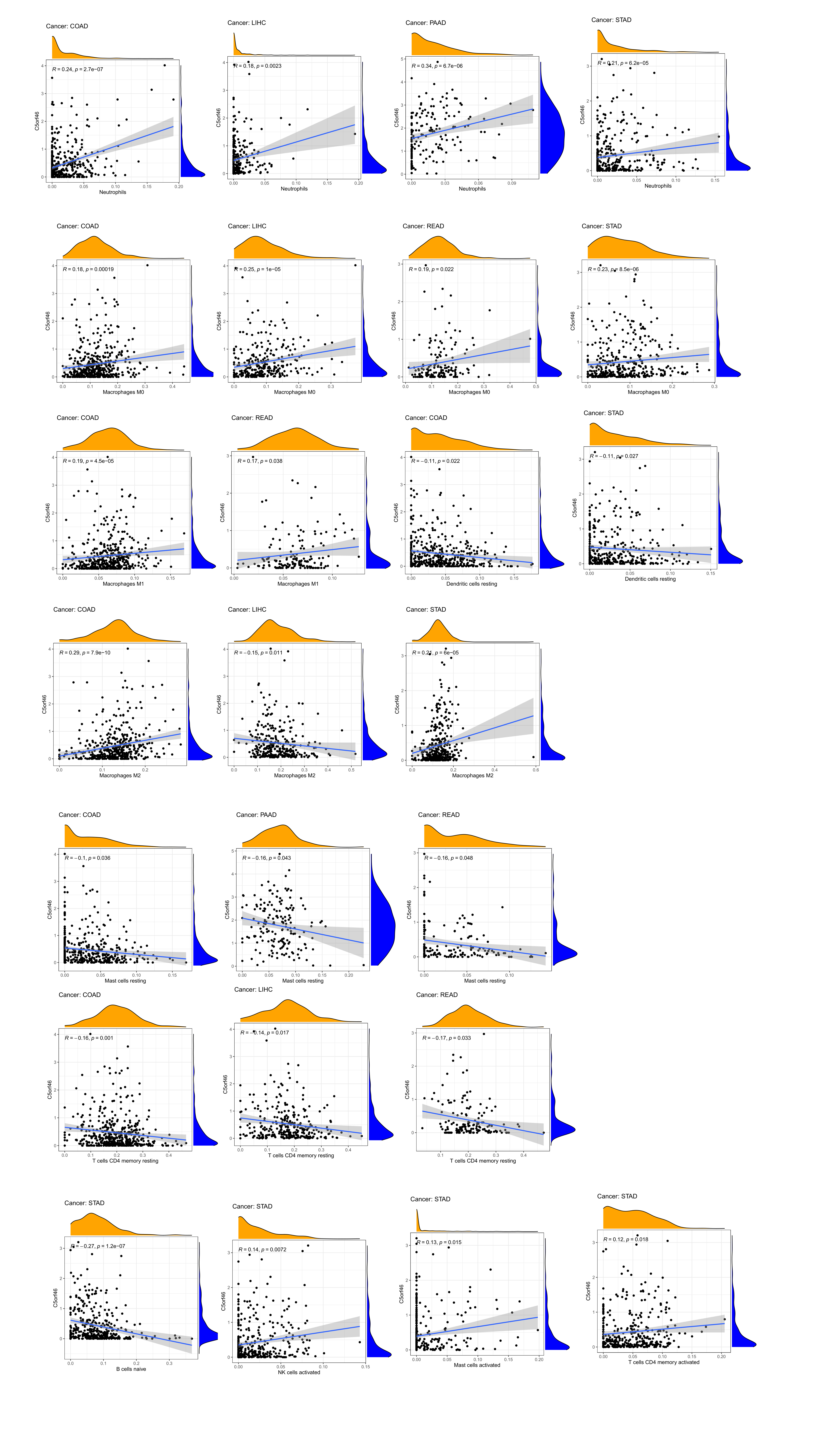

Supplement: Supplementary file 3 [file Image4.JPEG]

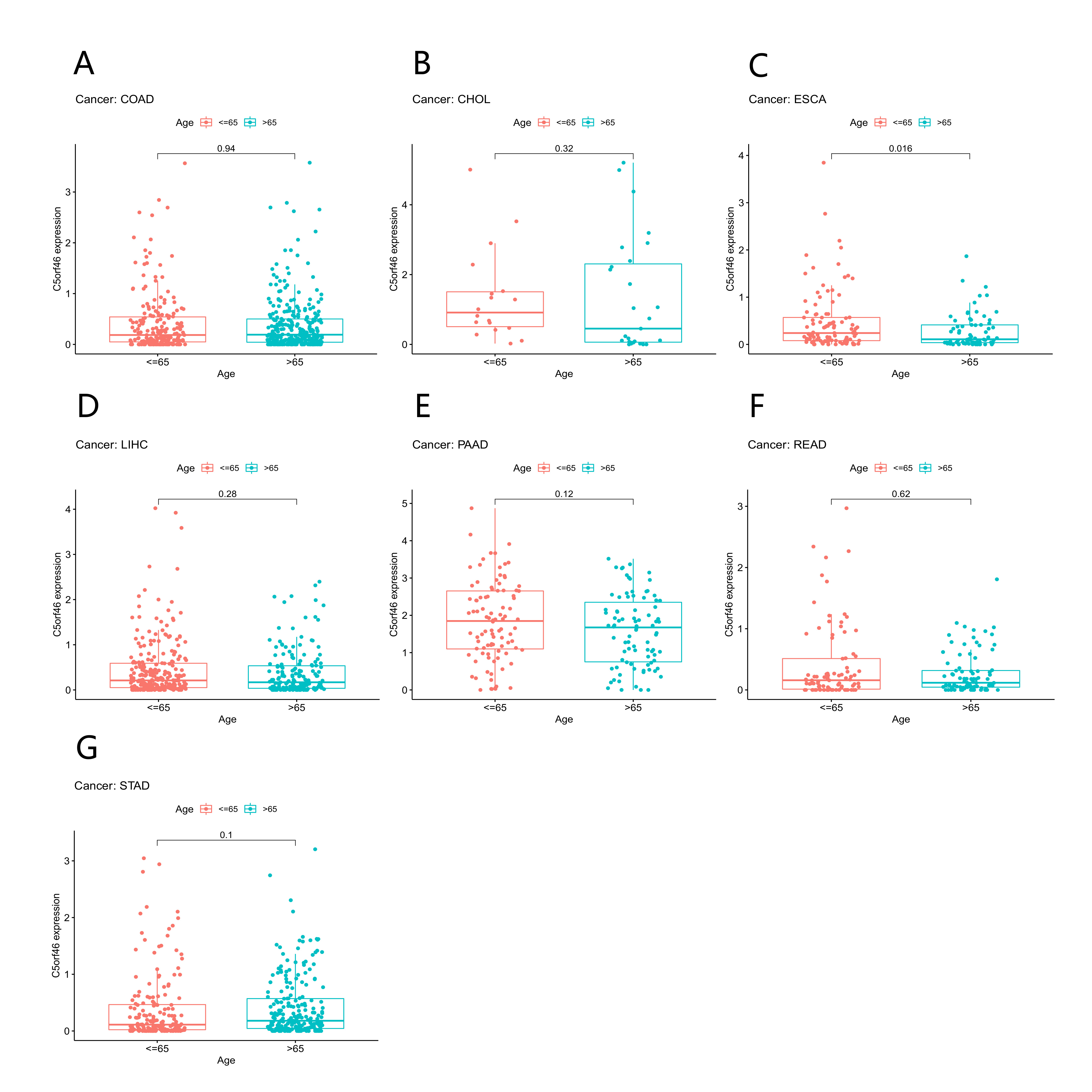

Supplement: Supplementary file 4 [file Image2.JPEG]
